# Astragaloside IV attenuates gestational diabetes mellitus via targeting NLRP3 inflammasome in genetic mice

**DOI:** 10.1186/s12958-019-0522-7

**Published:** 2019-09-26

**Authors:** Ruixue Zhang, Xuelei Zhang, Baoheng Xing, Jianyong Zhao, Peipei Zhang, Dandan Shi, Fengzhen Yang

**Affiliations:** 1 Cangzhou Central Hospital, No. 16 Xinhua West Road, Cangzhou, 061000 Hebei China; 2 Cangzhou Hospital of Integrated TCM-WM, HEBEI, No. 31, Huanghe Road, Cangzhou, 061000 Hebei China

**Keywords:** Astragaloside IV, Gestational diabetes mellitus, NLR family pyrin domain containing-3, Inflammasome

## Abstract

**Background:**

As the most ordinary metabolic disorder during pregnancy, gestational diabetes mellitus (GDM) has become a severe risk for the health of both pregnant female and fetus. Astragaloside IV (AS-IV) is the dominant active component in *Astragalus membranaceus.* It has been proved that AS-IV has anti-inflammation and immune-regulation function. We aimed to demonstrate the function of AS-IV in the therapy of GDM and the molecular mechanism in this process.

**Methods:**

C57BL/KsJ-Lepdb/+ female mice were used as GDM model. The mRNA levels of relative genes in this research were detected by qRT-PCR. The protein levels of relative genes were analyzed by western blot. Serum concentration of interleukin-6 (IL-6) and tumor necrosis factor alpha (TNF-α) were analyzed by ELISA.

**Results:**

Glucose and insulin levels in GDM mice model were decreased by AS-IV treatment. AS-IV down-regulated the expression of inflammatory gene IL-6 and TNF-α in GDM mice model. AS-IV treatment inhibited the expression of NLR family pyrin domain containing-3 (NLRP3) inflammasome relative proteins in the pancreas of GDM mice.

**Conclusion:**

This study demonstrated that AS-IV treatment has an effective therapeutic function of GDM in mice model through the inhibition of NLRP3 inflammasome in the pancreas.

## Background

In a part of pregnant women, diabetes is promoted by pregnancy related hyperinsulinemia and insulin resistance (IR) [1]. Gestational diabetes mellitus (GDM) is a metabolic disorder in pregnant female which is characterized by the glucose intolerance during the second or third trimester of pregnancy [2]. In pregnant women, the adverse outcomes caused by the pathogenesis of GDM includes insulin resistance, hyperinsulinemia, hyperglycemia, and abnormal embryo development [3]. The disorder caused by GDM during the embryo development includes stillbirth, metabolic disturbances, and fetal macrosomia [4]. GDM will cause diabetes after pregnancy in nearly 5% of patients and its pathogenesis is hard to be predicted since it has nearly no prior signs [5]. The increased GDM incidence is promoted by the increased incidence of obesity and the increasing maternal age [6]. It has been demonstrated that the pathogenesis of GDM is triggered by environmental and genetic factors simultaneously, but the molecular mechanism during this process is still not clear [7]. In GDM patients, the insulin secretion is decreased by promoted insulin resistance rate [8]. Previous studies have proved the connection between the activation of the NLRP3 inflammasome and insulin resistance [9]. Since GDM has become a severe risk for the health of both mother and baby, the efficacious therapeutic strategy of GDM is needed urgently. C57BL/KsJ-Lepdb/+ (db/+) mouse is proved to be a great GDM animal model since it can mimic most of GDM symptoms in pregnant women and fetus [10].

*Astragalus membranaceus* is a common medicine in Chinese traditional medicine which is proved to play a role in the therapy of a variety of diseases, such as hepatitis, kidney disease, and cardiovascular disease [11]. Among the extraction of *Astragalus membranaceus*, Astragaloside IV (AS-IV) is the dominant active component [12]. AS-IV has been confirmed to be a multifunction molecule since it has functions including anti-inflammation, anti-oxidative activity, anti-cardiac hypertrophy, anti-cerebral edema, and anti-diabetes [13–17]. The pathogenesis of GDM has strong correlation with the abnormal inflammation response during the pregnancy [18]. As a consequence of its anti-inflammation function, AS-IV may play a role in the therapy of GDM.

In this research, we aimed to explore the function of AS-IV in the therapy of GDM in mice model and demonstrate the molecular mechanism in this process.

## Methods

### Animals

C57BL/KsJ-Lep+/+ mice (wild type) and C57BL/KsJ-Lepdb/+ (db/+) mice were purchased from Jackson Laboratories. The C57BL/KsJ-Lepdb/+ mouse had a mutation in the gene encoding the leptin receptor, and leptin deficiency confers susceptibility to obesity, insulin resistance, and type 2 diabetes mellitus (T2DM). C57BL/KsJ-Lepdb/+ mice were currently the most widely used mouse model of T2DM, and were adapted in this study to establish GDM. Pregnant GDM mice were divided into three groups: Vehicle group (administered by oral gavage, water), AS-IV (low) group (administered by oral gavage, 15 mg/kg), and AS-IV (high) group (administered by oral gavage, 30 mg/kg). To investigate the underlying mechanism of AS-IV, pregnant GDM mice were also divided into three groups: Control group (administered by oral gavage, water), AS-IV group (administered by oral gavage, 30 mg/kg), and Glyburide group (administered by oral gavage, 50 mg/kg). Glyburide is an inhibitor of NLRP3 inflammasome, and was selected as positive control. Mice were treated daily. Animal studies were approved by the ethics commitment of Cangzhou Central Hospital.

### Serum glucose and insulin level measurement

Serum glucose and insulin levels were analyzed on gestation day (GD) 0, 7, and 14. Glucometer (Lifescan Surestep) was employed to detect the glucose level in serum. UltraSensitive Mouse Insulin ELISA kit (ALPCO Diagnostics) was used to analyze the serum insulin level.

### Glucose tolerance test

Mice on GD 14 were fasted for 6 h and treated with 2 g/kg body weight glucose through intraperitoneal injection. Blood samples were collected from the tail using capillary tubes at 0, 30, 60, and 90 min after glucose supplement.

### Insulin tolerance test

Mice on GD 14 were fasted for 6 h and treated with 0.75 U/kg body weight insulin through intraperitoneal injection. Blood samples were collected from the tail using capillary tubes at 0, 30, 60, and 90 min after insulin treatment.

### Cytokine assay

IL-6 and TNF-α levels in serum of mice were measured by ELISA kit (Thermo Fisher, Waltham, MA, USA) under the instructions from manufacturer.

### Quantitative real-time PCR (qRT-PCR)

The pancreas total RNA was isolated by Tri-reagent based on the manufacture’s instruction. High Capacity cDNA Reverse Transcription kit was used in the reverse transcription of cDNA from total RNA. qRT-PCR was performed using SYBR green master mix in triplicates. Primers used in this experiment were shown as follows:

NLRP3 sense 5′-TACGGCCGTCTACGTCTTCT-3′;

NLRP3 antisense 5′-CGCAGATCACACTCCTCAAA-3′;

Caspase-1 sense 5′-CACAGCTCTGGAGATGGTGA-3′;

Caspase-1 antisense 5′-TCTTTCAAGCTTGGGCACTT-3′;

IL-1β sense 5′-CAACCAACAAGTGATATTCTCCATG-3′;

IL-1β antisense 5′-GATCCACACTCTCCAGCTGCA-3′;

β-actin sense 5′-TCGCTGCGCTGGTCGTC-3′;

β-actin antisense 5′-GGCCTCGTCACCCACATAGGA-3′.

### Western blot

Western blot was performed with the standard method. The following antibodies were used: IL-1β (Cell Signaling Technology, MA, USA); NLRP3 (Cell Signaling Technology, MA, USA); Caspase-1 (Abcam, Cambridge, UK); IL-6 (Abcam, Cambridge, UK); TNF-α (Abcam, Cambridge, UK); β-actin (Cell Signaling Technology, MA, USA).

### Statistical analyses

SPSS software version 19.0 was used in the data analyses. All data in the experiment was shown as mean ± SD. One- or two-way ANOVA analysis followed by a Bonferroni post hoc test was used to calculate the differences between the experimental groups.

## Results

### AS-IV attenuated GDM symptoms in pregnant db/+ mice

**Fig. 1:**
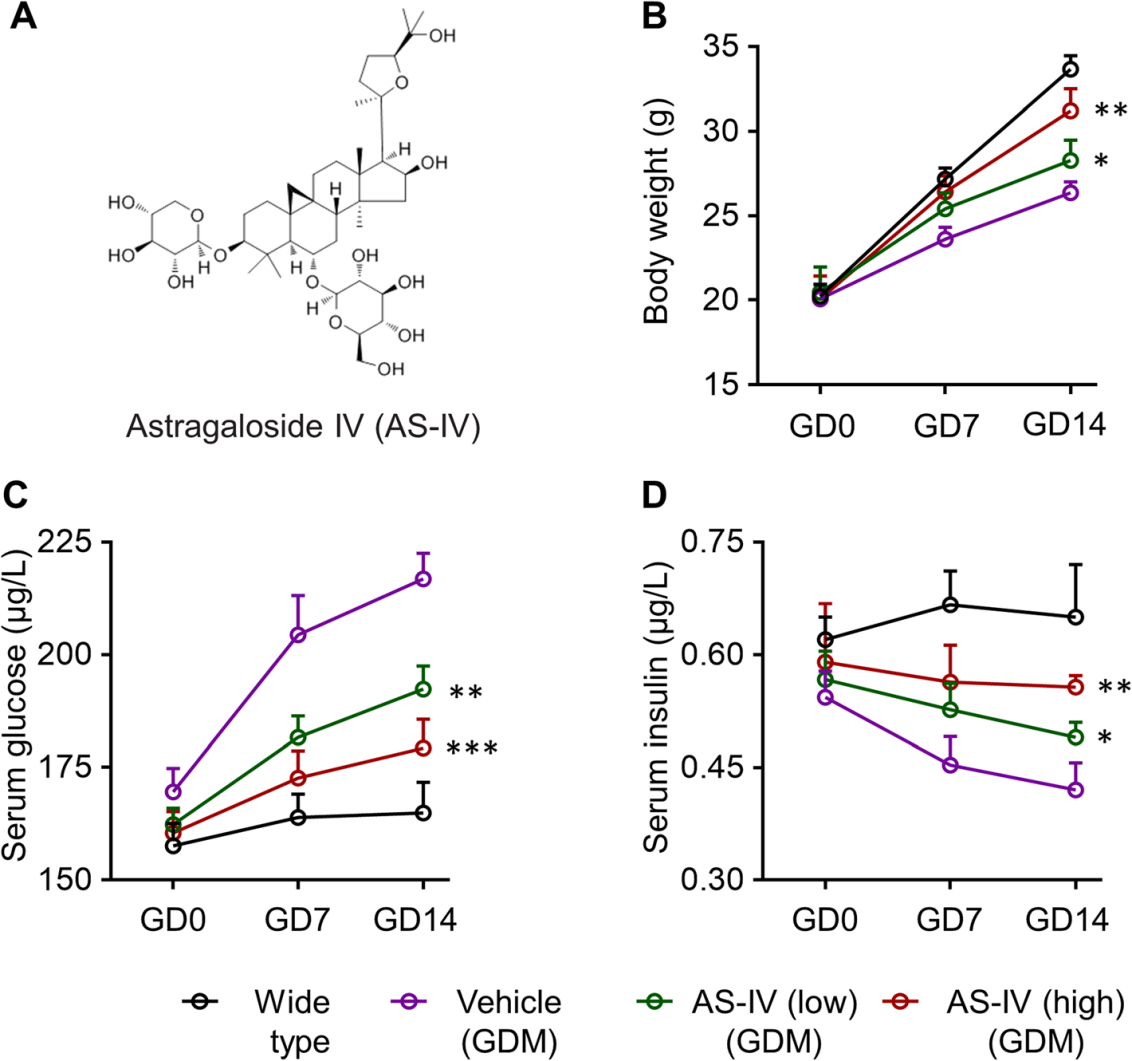
Astragaloside IV (AS-IV) attenuates gestational diabetes mellitus (GDM) symptoms in GDM mice. **a** Chemical structure of Astragaloside IV (AS-IV). Maternal body weight (**b**), serum glucose level (**c**) and insulin level (**d**) were measured on gestation day (GD) 0, 7 and 14 in wild-type group, GDM groups treated with vehicle or AS-IV. Pregnant GDM mice were divided into three groups: Vehicle group (orally gavaged with water), AS-IV (low) group (orally gavaged with AS-IV, 15 mg/kg) and AS-IV (high) group (orally gavaged with AS-IV, 30 mg/kg), mice were treated daily and blood samples were collected from the tails using capillary tubes for examination. Data were presented as mean ± SD. *n* = 8. * *p* < 0.05, ** *p* < 0.01, *** *p* < 0.001, vs vehicle-treated GDM mice

We first examined the effect of AS-IV (chemical structure shown in Fig. 1a) treatment on the GDM symptoms in pregnant db/+ mice. It was demonstrated that, compared with wild type mice, maternal body weight was decreased in GDM model mice (Fig. 1b). AS-IV treatment significantly increased the maternal body weight of pregnant db/+ mice in a dose-dependent manner, compared to wild type mice (Fig. 1b). Hyperglycemia and insulin resistance are two dominant symptoms in GDM. In GDM patients, the insulin secretion is decreased by promoted insulin resistance rate [8]. Then we analyzed the effect of AS-IV treatment in the levels of glucose and insulin in serum of GDM model mice. Based on our result, AS-IV treatment significantly reduced the elevated glucose levels in serum of db/+ mice compared with wild type mice (Fig. 1c). Meanwhile, the declined insulin levels in db/+ mice serum were up-regulated by AS-IV treatment when compared with the control group (Fig. 1d).

### AS-IV treatment improved reproductive outcome of pregnant db/+ mice

**Fig. 2:**
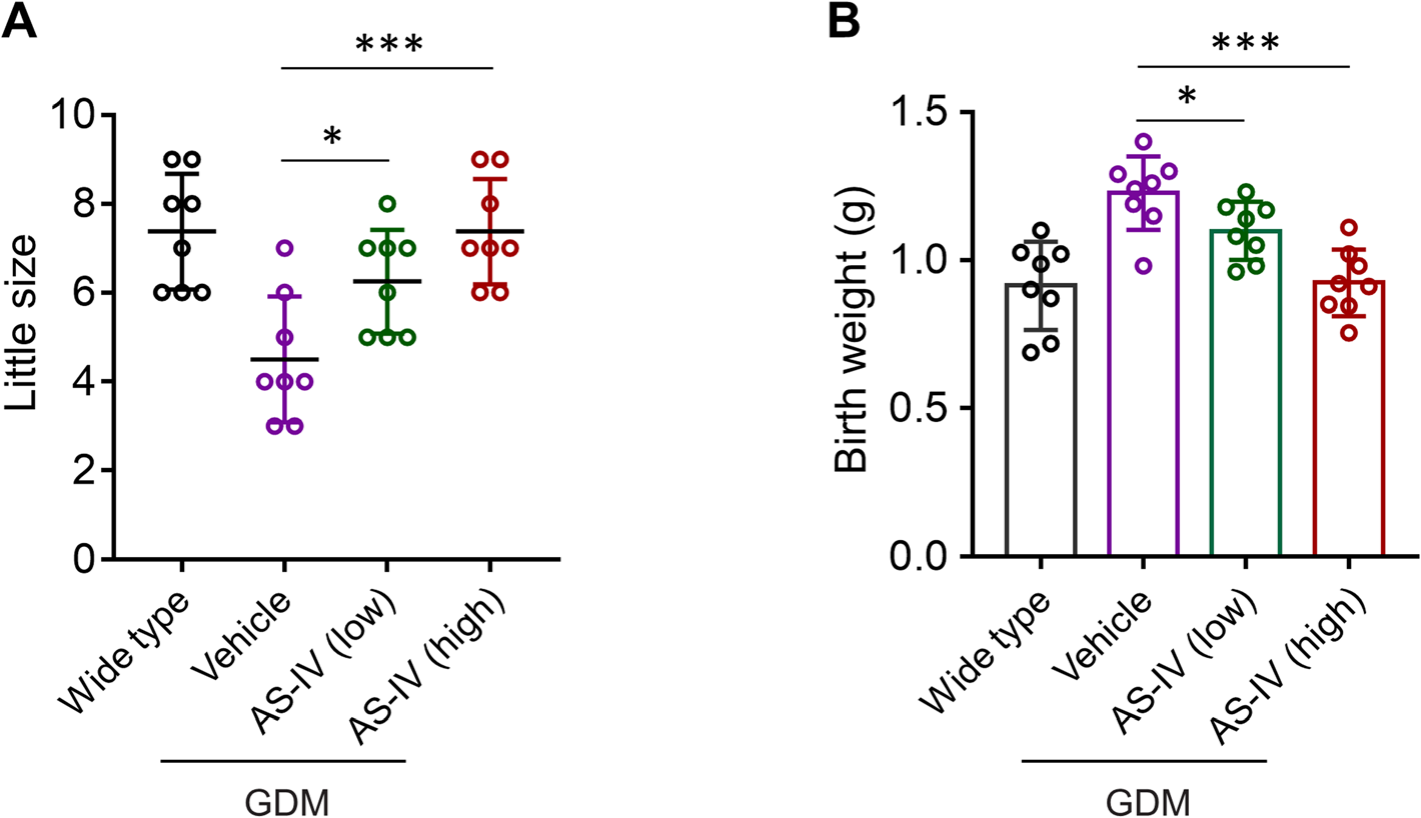
AS-IV treatment improved reproductive outcome of pregnant GDM mice. Litter size (**a**) and body weight at birth (**b**) of offspring born by each female mouse from wild type and AS-IV-treated GDM mice. Data was presented as mean ± SD. *n* = 8, * *p* < 0.05, *** *p* < 0.005, vs vehicle-treated GDM mice

Since AS-IV treatment attenuated GDM symptoms in pregnant female mice, we investigated whether AS-IV was able to rescue the disorder in the offspring. In fetuses, the adverse outcomes caused by GDM include decreased fetus number and increased offspring body weight [19]. The number of offspring as birth for each pregnant mouse in four different groups was counted (Fig. 2a). The decreased litter size in Vehicle group demonstrated the decreased fetal survival in db/+ mice. In contrast, the treatment of AS-IV in GDM mice model significantly increased the offspring number in a dose-dependent manner. We also analyzed the body weight of offspring at birth in four different groups (Fig. 2b). Based on our data, the elevated mean body weight of fetus in db/+ mice was rescued by AS-IV treatment. These data proved that AS-IV played a role in the improvement of reproductive outcome in GDM mice model.

### AS-IV treatment inhibited inflammation in GDM mice model

**Fig. 3:**
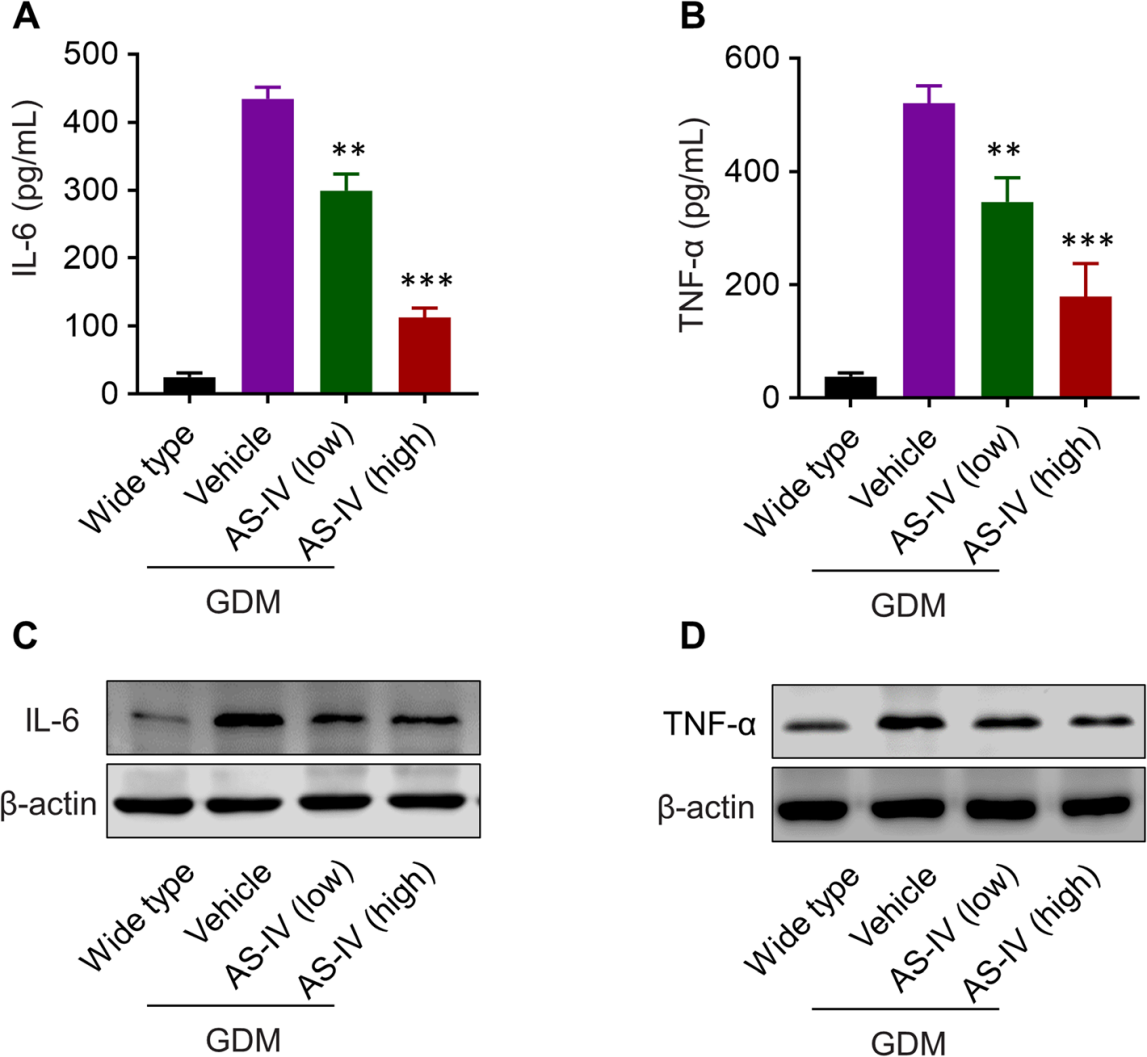
AS-IV treatment inhibited inflammation in GDM mice model. Serum concentration of IL-6 (**a**) and TNF-α (**b**) in pregnant wild type and AS-IV-treated GDM mice were measured on gestation day 14 via ELISA. Protein expression of IL-6 (**c**) and TNF-α (**d**) in the pancreas of pregnant wild mice or treated GDM mice were detected by western blotting. β-actin was set as control protein. Data was presented as mean ± SD. *n* = 8. ** *p* < 0.01, *** *p* < 0.005, vs vehicle-treated GDM mice

During the pathogenesis of GDM, the level of proinflammatory cytokine TNF-α and IL-6 are elevated by hyperglycemia caused disorder in inflammation and oxidative stress [20, 21]. To illustrate whether AS-IV has influence on the level of proinflammatory cytokines in GDM mice model, the serum level of TNF-α and IL-6 were measured by ELISA (Fig. 3a, b). The serum level of TNF-α and IL-6 were elevated in GDM mice model when compared with wild type mice. After the treatment, the expression of TNF-α and IL-6 in the serum of db/+ mice were dramatically down-regulated by AS-IV in a dose-dependent manner. Furthermore, we also analyzed the protein level of TNF-α and IL-6 in the pancreas of pregnant mice (Fig. 3c, d). Based on the western blot results, the increased protein level of TNF-α and IL-6 in pancreas were also down-regulated through the treatment of AS-IV in pregnant db/+ mice. Based on these data, AS-IV rescued the activated inflammatory response in GDM mice model.

### AS-IV treatment inhibited NLRP3 inflammasome in the pancreas of GDM mice model

**Fig. 4:**
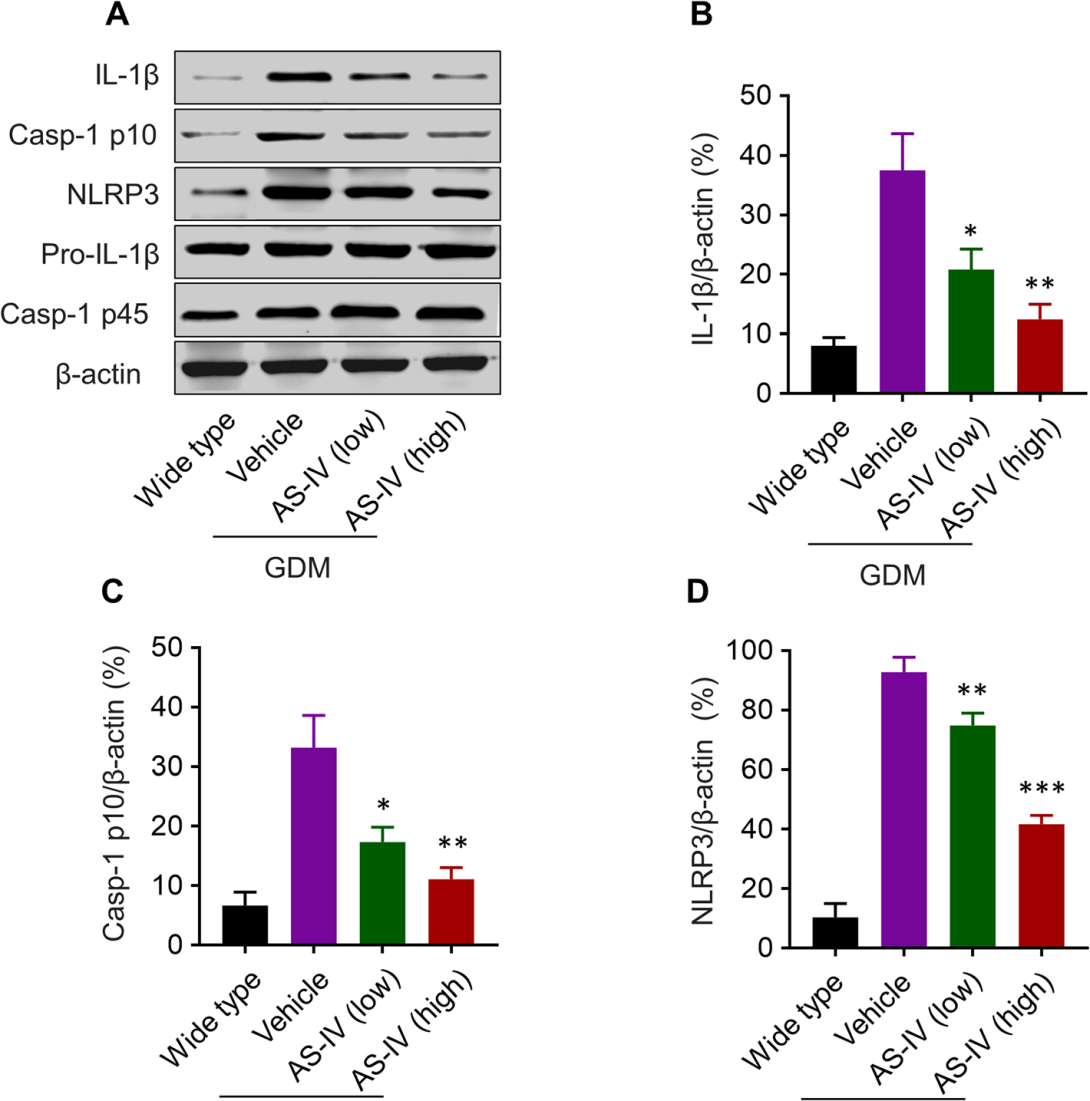
AS-IV treatment inhibited NLRP3 inflammasome in the pancreas of GDM mice model. **a** Detection of inflammasome proteins expression (including IL-1β, Casp-1 p10, NLRP3, Pro-IL-1β, and Casp-1 p45) in the pancreas of pregnant wild mice or treated GDM mice on gestation day 14. β-actin was set as control protein. Relative expression of IL-1β (**b**), Casp-1 p10 (**c**), and NLRP3 (**d**) compared to control protein β-actin. Data was presented as mean ± SD. *n* = 8. * *p* < 0.05, ** *p* < 0.01, *** *p* < 0.005, vs vehicle-treated GDM mice

Since the activation of the NLRP3 inflammasome has a critical function in insulin resistance, we analyzed the level of inflammasome relative proteins in the pancreas of pregnant mice (Fig. 4a). The formation of NLRP3 inflammasome leads to the self-cleavage of pro-Caspase-1 (Casp-1 p45), generating the active Caspase-1 (Casp-1 p10) [22]. Active Caspase-1 induces the conversion of immature IL-1β (Pro-IL-1β) into active IL-1β [22]. We analyzed the changes in the protein level of IL-1β, Casp-1 p10, and NLRP3 in four different groups (Fig. 4b, c, d). The higher IL-1β, Casp-1 p10, and NLRP3 levels in db/+ mice than wild type illustrated the activation of inflammasomes in pancreas tissue of GDM mice model. The protein level of IL-1β, Casp-1 p10, and NLRP3 were all declined by the treatment of AS-IV in the pancreas of GDM mice model in a dose-dependent manner. So NLRP3 inflammasome was inhibited by AS-IV in the pancreas of GDM mice model.

### AS-IV had the same function as NLRP3 inhibitor glyburide in the treatment of GDM in mice

**Fig. 5:**
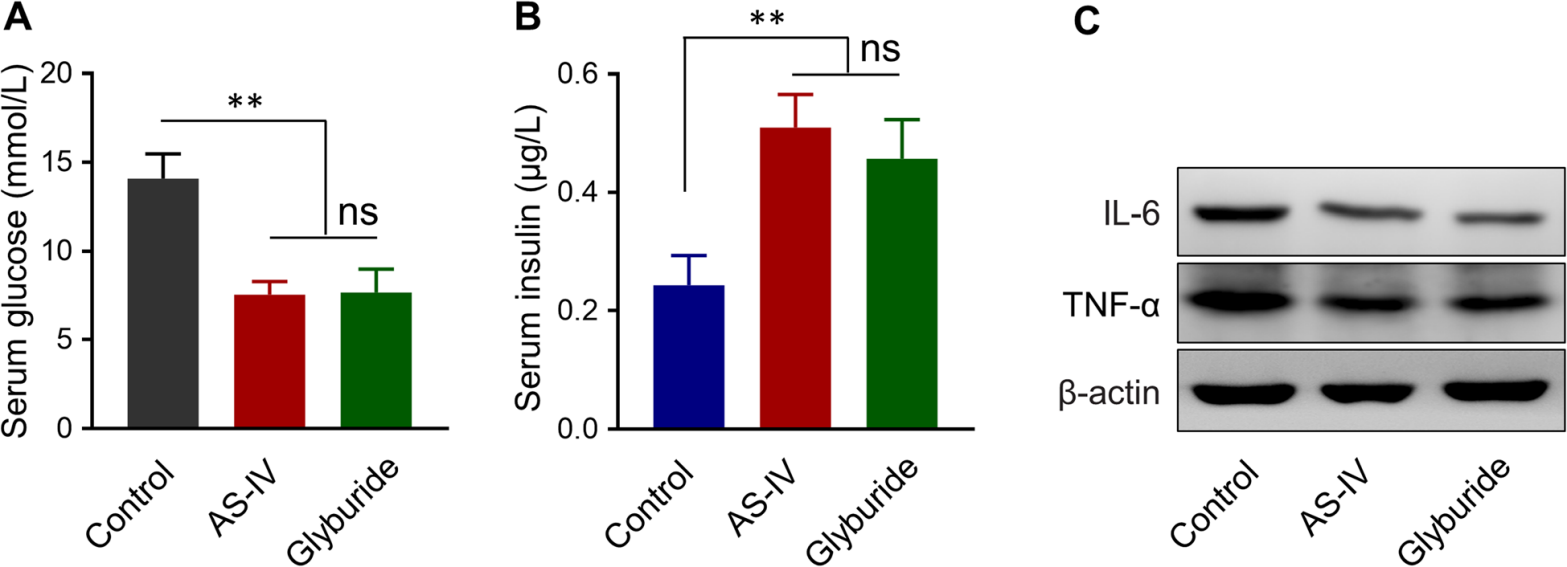
Effect of AS-IV and NLRP3 inhibitor on GDM symptoms in mice model. Serum concentration of glucose (**a**) and insulin (**b**) in pregnant GDM mice treated with AS-IV or glyburide (a specific inhibitor of NLRP3) were measured on GD14. **c** Protein levels of IL-6 and TNF-α in the pancreas of pregnant GDM mice treated with AS-IV or glyburide. β-actin was set as control protein. Data was presented as mean ± SD. *n* = 8. **p < 0.01, ns, no significant difference

NLRP3 inhibitor glyburide has become the most prescribed medication for GDM [23]. Since AS-IV also had NLRP3 inflammasome inhibition function, we aimed to illustrate whether AS-IV had the same function as glyburide in the attenuation of GDM symptoms in pregnant db/+ mice. The elevated serum glucose level in GDM mice model was decreased by both AS-IV and glyburide treatment (Fig. 5a). The decreased serum insulin level in GDM mice model was up-regulated by both AS-IV and glyburide treatment (Fig. 5b). Based on the western blot results, the protein level of TNF-α and IL-6 in db/+ mice were down-regulated by both AS-IV and glyburide treatment (Fig. 5c). All these data proved that AS-IV had the same function as glyburide in the therapy of GDM in mice.

## Discussion

AS-IV is the dominant active component in *Astragalus membranaceus*, a Chinese traditional medicine [12]. As a multifunction molecule, AS-IV has been reported to participates in the therapy of several different diseases through its anti-inflammation and anti-oxidant abilities. As a lethal complication of diabetes mellitus, diabetic nephropathy is ameliorated by AS-IV through the inhibition of extracellular signal-regulated kinase 1 and 2 (Erk1/2), nuclear factor kappa B (NFκB), and protein kinase B (Akt)/ mammalian target of rapamycin (mTOR) signaling pathways in mice model [15]. Diabetic retinopathy in type 2 diabetic (T2DM) db/db mice is prevented by AS-IV through the inhibition of aldose reductase activity and ERK1/2 phosphorylation [24]. Based on the previous studies in the mechanism of GDM pathogenesis, GDM can be defined as the early stage of T2DM which develops in the second and third trimester of pregnancy [25]. GDM patients have a higher risk to develop T2DM after pregnancy, while the fetuses also have a higher T2DM and obesity incidence in their early life [26]. Since the treatment of AS-IV has shown the benefits in the therapy of several complications of diabetes in mice, we aimed to illustrate whether AS-IV plays a role in the therapy of GDM in mice model.

During late gestation (third trimester), GDM patients have insulin resistance and lower insulin secretion rates and glucose uptake rates [8]. The decreased insulin secretion and the enhanced insulin-resistant state disturb the maternal insulin balance then trigger the maternal glucose intolerance and promote the pathogenesis of GDM [27]. To illustrate the effect of AS-IV in the therapy of GDM in db/+ genetic mice model, we first examined the level of glucose and insulin in serum. The increased glucose level in the serum of GDM mice model was significantly declined by AS-IV treatment in a dose-dependent manner. Meanwhile, down-regulated serum insulin level caused by lower insulin secretion rates in GDM was up-regulated by AS-IV. The altered glucose and insulin levels in the serum of GDM mice model by AS-IV demonstrated the function of AS-IV in the alleviation of the symptoms of GDM in db/+ mice.

GDM also generates several adverse outcomes in fetuses, such as stillbirth, metabolic disturbances, and fetal macrosomia [4]. In this research, we detected the maternal reproductive status of each group to further confirm the effect of AS-IV in the therapy of GDM in mice model. Macrosomia (enlarged fetus size) in GDM is caused by hyperglycemia promoted hyperinsulinemia in offspring [28]. The body weight at birth of offspring in AS-IV treated groups were significantly reduced when compared with the control group. The elevated intrauterine fetal death rate is observed in pregnant women with GDM and causes the decrease in offspring number [29]. In this research, the decrease in the number of live fetuses was observed in db/+ mice when compared with wild type mice. On the other side, AS-IV treatment elevated the little size of db/+ mice in a dose-dependent manner. The improved maternal reproductive status in GDM mice model by AS-IV confirmed the function of AS-IV in the alleviation of the symptoms of GDM in db/+ mice.

During the pathogenesis of GDM, the level of pro-inflammatory cytokine TNF-α and IL-6 are elevated by hyperglycemia caused disorder in inflammation and oxidative stress [20, 21]. The acute pro-inflammatory response triggered by the disorder in metabolites causes systemic insulin resistance [30]. It has been proved that the placental inflammation in the pathogenesis of GDM is crucial in determining the fetal environment during pregnancy [31]. In this research, we checked the level of pro-inflammatory cytokine TNF-α and IL-6 in both pancreas and serum. The declined level of TNF-α and IL-6 in both pancreas and serum of db/+ mice illustrated the function of AS-IV in the regulation of acute pro-inflammatory response in GDM.

Although insulin resistance is crucial in the pathogenesis of GDM, the mechanism in the initiation of insulin resistance is still not clear. It is reported that the down-regulated insulin signaling in GDM is regulated by the pro-inflammatory cytokines generated from adipose tissue [32]. IL-1β is one of the dominant cytokines which participates in the regulation of insulin signaling in GDM [33]. The active IL-1β is generated from pro IL-1β by the active Caspase-1 and the Caspase-1 is activated through NLRP3 inflammasome [34]. The activation of caspase-1 enhances the secretion of IL-1β from adipose tissue in GDM and triggers the development of insulin resistance [35].

AS-IV has been reported to has neuroprotective effects against transient cerebral ischemia through the suppression of NLRP3 inflammasome activation [36]. So, we aimed to explore whether AS-IV can inhibit the NLRP3 inflammasome in GDM mice model. To answer this question, we analyzed the protein level of NLRP3, Caspase-1, and IL-1β through western blot. In pancreas of db/+ mice, the elevated protein level of NLRP3, Caspase-1, and IL-1β were all decreased after AS-IV treatment in a dose-dependent manner. These data demonstrated that AS-IV inhibited the NLRP3 inflammasome in the pancreas of GDM mice model.

To demonstrate the function of AS-IV in the alleviation of the symptoms of GDM is based on the inhibition of NLRP3 inflammasome, we also treated the db/+ mice with NLRP3 inhibitor glyburide. The treatment of glyburide shown the same function of AS-IV in the alleviation of GDM symptoms including hyperglycemia, insulin resistance, and activated inflammatory response. So, the NLRP3 inflammasome inhibition function was crucial for AS-IV in the therapy of GDM in mice model. However, we found high injection dosage of AS-IV had maternal toxicity, as evidenced by the fact that there were more pregnant mice with dead litters and higher mortality, compared with control group. When the dose of AS-IV was 15 mg/kg (orally), no significant maternal toxicity was observed (Additional file 1: Table S1). These results indicated that the dosage of AS-IV and routes of administration should be carefully considered during pregnancy.

## Conclusion

In conclusion, we have demonstrated that AS-IV is efficacious in alleviation of GDM symptoms in mice model through the inhibition of NLRP3 inflammasome. This result provides us a new strategy for the prevention and therapy of GDM in human.

## Supplementary information

**Additional file 1: Table S1.** Reproductive parameters in mice administered astragaloside IV.

## Data Availability

All data generated or analysed during this study are included in this published article.

## Abbreviations

AS-IV: Astragaloside IV
GDM: gestational diabetes mellitus
IL-6: interleukin-6
TNF-α: tumor necrosis factor alpha
