# Supplementary material for: Astragaloside IV attenuates gestational diabetes mellitus via targeting NLRP3 inflammasome in genetic mice
Source: Reprod Biol Endocrinol. 2019 Sep 26;17:77. doi: 10.1186/s12958-019-0522-7 (PMC6764134; doi:10.1186/s12958-019-0522-7)
Supplement: Supplementary file 1 — Additional file 1: Table S1. Reproductive parameters in mice administered astragaloside IV. [file 12958_2019_522_MOESM1_ESM.docx]

Additional file 1: Table S1. Reproductive parameters in mice administered astragaloside IV

|  | Control | Astragaloside IV (15 mg/kg) | Astragaloside IV (30 mg/kg) |
| --- | --- | --- | --- |
| Number (%) of pregnant females | 10/15 (66.7) | 10/15 (66.7) | 9/15 (60.00) |
| Number of corpora lutea (mean ± SD) | 92 | 89 | 95 |
| Number of implantation sites | 85 | 86 | 83 |
| Number of live fetuses (%) | 74 (87.0) | 71 (82.6) | 69 (83.1) |
| Number of females with resorptions (%) | 4 (4.7) | 3 (3.4) | 6 (7.2) |
| Number of with resorptions/litters (%) | 1/10 (10) | 2/10 (20) | 1/9 (11.1) |
| Number of dead fetuses (%) | 7 (8.2) | 8 (9.3) | 11 (13.2) |
| Number of with dead litters (%) | 3/10 (30) | 3/10 (30) | 5/9 (55.5)* |
| Mortality (%) | 12.9 | 12.7 | 20.4** |
| Average fetal weight (g) | 0.87 ± 0.13 | 0.92 ± 0.17 | 0.89 ± 0.22 |
| Fetal sex rate, M/F | 0.98:1 | 1.09:1 | 1.09:1 |

Mortality: resorptions + dead fetuses.

**P* < 0.05; ***P* < 0.01. differences relative to control.
